# Venturing Into the Unknown: The Importance of Variable Selection When Modelling Alien Species Under Non‐Analogue Climatic Conditions

**DOI:** 10.1002/ece3.70490

**Published:** 2024-10-28

**Authors:** Tom Vorstenbosch, Franz Essl, Bernd Lenzner, Johannes Wessely, Stefan Dullinger

**Affiliations:** ^1^ Department of Botany and Biodiversity Research University of Vienna Vienna Austria; ^2^ Vienna Doctoral School of Ecology and Evolution University of Vienna Vienna Austria

**Keywords:** biological invasions, introduced plants, model transferability, novel climate, species distribution modelling, sub‐Antarctic Islands

## Abstract

Species distribution models (SDMs) are widely used to address species' responses to bioclimatic conditions in the fields of ecology, biogeography and conservation. Among studies that have addressed reasons for model prediction variability, the impact of climatic variable selection has received limited attention and is rarely assessed in sensitivity analyses. Here, we tested the assumption that this aspect of model design is a major source of uncertainty, especially when projections are made to non‐analogue climates. As a study system, we used 142 alien plant species introduced to the sub‐Antarctic islands. Based on global occurrence data, we fitted SDMs as functions of seven bioclimatic variable sets that only differed in the identity of two temperature variables. Moreover, we calculated the overlap between the island's climatic conditions and the niches the species have realised outside of the islands. Despite comparable internal evaluation metrics, projections of these models were in sharp contrast with each other, with some models predicting the sub‐Antarctic islands' climate to be almost ubiquitously suitable to most species and others unsuitable to almost all species. In particular, the mean temperature of the warmest month led to strong underpredictions of the SDMs, while its replacement by the mean temperature of the coldest month led to massive overpredictions. Partitioning the variance in projections demonstrated that predictor identity was its most important source, followed by island and species identity. The size of area projected to be suitable was also related to the overlap in predictor values realised in the global range of species (outside of the islands) and on the islands. Our findings emphasise the importance of bioclimatic variable selection in SDMs, especially when making projections to non‐analogue climates. Such extrapolations are often required, especially when using SDMs to assess invasion risk under both current and future climates.

## Graphical abstract

While having received less attention than other model parameters, variable selection can have a strong influence on predictions of species distribution models. This study shows how models with different sets of bioclimatic variables lead to widely varying predictions under non‐analogue conditions, using the sub‐Antarctic as a study system.

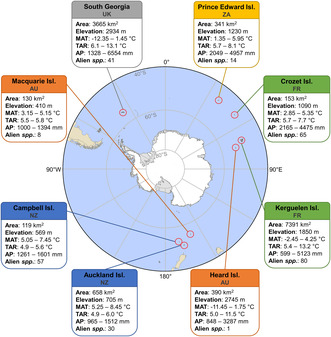

## 1 Introduction

The changing climate has triggered demand for scenarios of its environmental consequences, including the distribution of biota at local to global scales. Species distribution models (SDMs), often also called ecological niche models, have been by far the most popular approach for such scenarios over the last two decades. These models are popular because they have a sound theoretical foundation in the concept of the ecological niche, the data needed for their parameterisation is comparatively easy to collect and software to implement them is readily available. However, there are also well‐known sources of uncertainty associated with their predictions. While some of these sources have received considerable attention already, such as conceptual issues (e.g. Jiménez‐Valverde, Lobo, and Hortal 2008; Early and Sax 2014), neglect of processes (Wisz et al. 2013; Urban et al. 2016), use of different modelling algorithms (Dormann et al. 2008; Barbet‐Massin et al. 2012; Valavi et al. 2022) and emission scenarios (Steen et al. 2017; Thuiller et al. 2019), others have been explored less intensively. Among the latter is the selection of climatic variables as predictors in the models, a decision that comes with a brief or even no justification in many SDM applications and which is rarely included in sensitivity analyses. In fact, the lack of such sensitivity analyses is surprising given that a few case studies, which specifically focused on the impact of predictor choice, have indicated a potentially large effect on modelling outcomes, especially on projections to future climatic conditions (Synes and Osborne 2011; Braunisch et al. 2013).

It is well‐known that projections of SDMs are particularly problematic when they are made onto conditions not represented in the data used to parameterise the model, so‐called non‐analogue conditions, especially if the parameterisation data do not capture the full realised niche of a species (‘truncated niche’, e.g. Guisan, Thuiller, and Zimmermann 2017). However, such exceedance of currently realised variable values is only one kind of non‐analogue condition. Less obvious but potentially equally influential is the change in relationships between variables, especially among those that are currently highly correlated. Such changes might occur, for example because summer and winter temperatures warm at different rates in the future or because the rates by which they warm vary independently across a study area. The problem in this case is that modellers often select one of the correlated variables and drop the other one(s) to avoid multicollinearity issues (Soley‐Guardia, Alvarado‐Serrano, and Anderson 2024). As long as both variables change at similar rates and correlations between them remain stationary, the particular selection will have little impact on projections. However, if rates of change are different and/or correlations are altered, projections will depend on which variable has been selected (Braunisch et al. 2013), at least if these variables are important in the model. Put differently, these easily overlooked aspects of non‐analogue climates can probably confer predictor choice a considerable impact on SDM projections. This impact will likely increase further if one of the two (currently) correlated variables will, in the future, exceed the value range currently realised in the parameterisation data but the other will not, for example if summer temperatures warm beyond those realised in the parameterisation data but winter temperatures do not. In such a case, the change in winter temperature might lead to projections of both rising or decreasing suitability, while the change in summer temperature should only lead to a decrease of projected suitability, at least if niches were fitted as non‐truncated functions of temperature. This is because (apparent) niche margins were identified in the currently realised range of summer temperature values, and hence exceedance of the latter will necessarily be classified as unsuitable conditions. More generally, the lower the overlap between variable values in the parameterisation data and the projection area, the lower the predicted suitability should be, by trend, at least as long as fitted niche models are not truncated.

The sub‐Antarctic islands offer an ideal empirical system to explore these assumed impacts of predictor choice under non‐analogue conditions without the need to make projections into an unknown future. The islands of the Southern Ocean are among the most isolated in the world. Climatic conditions are hyper‐oceanic with little seasonal and diurnal temperature variation, that is mild winters comparable to many temperate‐zone climates, but very cool summers that are typical for Arctic regions (Pendlebury and Barnes‐Keoghan 2007). In addition, rainfall is abundant throughout the year, including long periods of cloud cover and frequent strong winds (Fitzgerald and Kirkpatrick 2020). Similar combinations of climatic conditions are rare in other parts of the world. As a consequence, the climate of the islands is non‐analogue in comparison to most other places on Earth, even if the absolute values reached by individual climatic variables, such as summer and winter temperatures, are realised across smaller or larger areas elsewhere, too. Despite this non‐analogue climate, a considerable number of alien species has managed to establish on the islands, and a subset of them has even become invasive (Leihy et al. 2023) and exerts a substantial impact on the islands' native flora and fauna (Frenot et al. 2005; Greve et al. 2017). Although the islands have no permanent human populations in the present day, extensive whaling and sealing has taken place in the sub‐Antarctic during the 19th and 20th centuries (Headland 1989). This period saw the establishment of settlements on several of the sub‐Antarctic islands for which livestock, crops and grasses for pasture improvement were imported, along with many other species that came as stowaways (Bergstrom and Selkirk 2007). Whether the climate of the islands lies within the climatic niches these species have realised elsewhere in the world has not yet been evaluated. Given the peculiar combination of climate conditions on the island, we assume that the answer is ambiguous. In many species, the climate under which they grow outside of the island will broadly overlap with the one of the islands with respect to some variables, but for only a few this overlap will extend to all variables simultaneously. In particular, we expect that a broad overlap in both summer and winter temperatures at the same time is unlikely because they are rarely combined in this way elsewhere.

Following the assumption laid out above, we hence expect that SDMs calibrated with data on the global distribution of these species, with the exception of the islands, will deliver very different projections onto the non‐analogue conditions of the islands in dependence on the climatic variables used for model fitting. These differences will occur, although the accuracy of the models as evaluated with data from the parameterisation range, that is from outside the islands, will not differ substantially. We test these assumptions by fitting SDMs of 148 plant species reported as aliens from one or more of the islands using all native and non‐native occurrences from outside the islands and seven different sets of climatic predictor variables selected among those most commonly used in SDM applications. Subsequently, we partition the variation in the number of cells predicted suitable among the three sources of island, species and predictor variable set. We expect that the predictor set selected will have an impact on this variation comparable in magnitude to those of the other two sources. In addition, we expect that the number of cells predicted will systematically vary with how much the conditions on the island, as captured by the individual predictor set, overlap with the conditions in the species' geographical range outside of the island. In particular, we expect that a larger overlap will result in a larger number of cells predicted to be suitable.

## 2 Methods

### 2.1 Study Area

**FIGURE 1:**
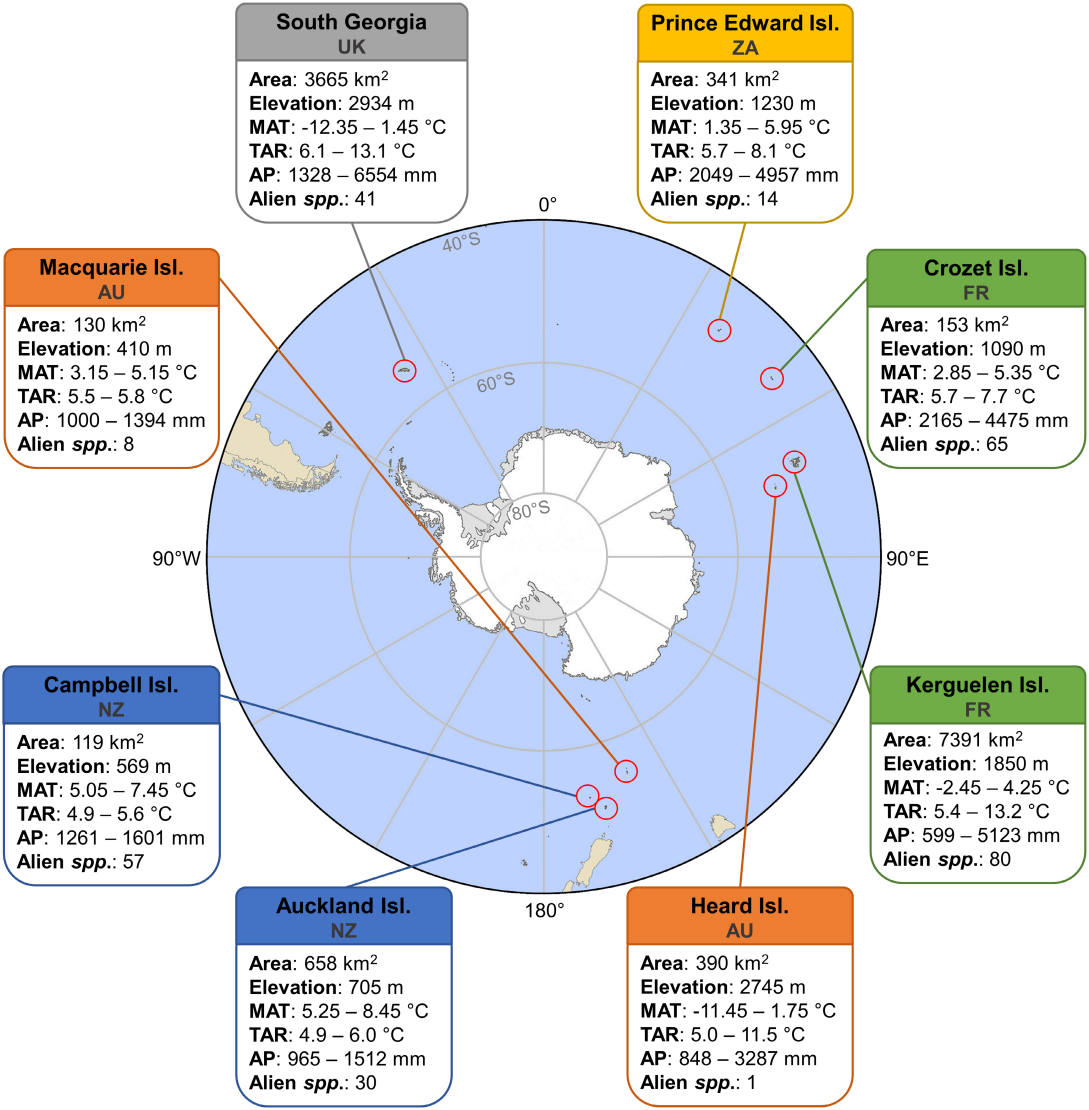
Map of the Southern Ocean and surrounding areas. Sub‐Antarctic archipelagos included in this study are shown with their territorial claims (AU = Australia; FR = France; NZ = New Zealand; UK = United Kingdom; ZA = South Africa), surface area (Area), elevation, mean annual temperature (MAT), temperature annual range (TAR), annual precipitation (AP) and number of alien species observed in vegetation surveys (alien spp.) (Leihy et al. 2023).

The study region encompasses eight archipelagos in the Southern Ocean, roughly located between 47° S and 54° S (Figure 1). The archipelagos constitute overseas territories of Australia (AU), France (FR), New Zealand (NZ), South Africa (ZA) and the United Kingdom (UK) and include the Auckland Islands (NZ), the Campbell Islands (NZ), the Crozet Islands (Possession Island, FR), Heard Island (AU), the Kerguelen Islands (FR), Macquarie Island (AU), the Prince Edward Islands (ZA) and the South Georgia Island group (UK). We excluded sub‐Antarctic islands with no reported alien plant introductions (e.g. Bouvet Island; the South Sandwich Islands) from the study. We did not consider the Falkland Islands in this study, as year‐round inhabitation accompanying horticulture makes the distinction between cultivated and escaped plants more difficult than on the other islands (Armstrong 1994).

### 2.2 Climatic Data

We obtained raster layers of 19 bioclimatic variables with a spatial resolution of 30 arc sec (~1 km) from the CHELSA climate world database, version 2.1, for the period 1981–2010 (Karger et al. 2017). These constitute a set of bioclimatic variables representing temperature and precipitation commonly used in SDMs (Guisan and Zimmermann 2000). We chose the bioclimatic variables provided by CHELSA because the underlying basic algorithm incorporates orographic predictors, which perform well in predicting rain shadows that likely are an important factor on the islands (Karger et al. 2020). The descriptions and value ranges of all bioclimatic variables for each island group are listed in Data S1. These ranges represent the lowest and highest values for each bioclimatic variable between any of the grid cells that cover the islands' land surface area averaged between 1981 and 2010.

### 2.3 Alien Flora of the Sub‐Antarctic Islands

We used the list of introduced species in the (sub‐)Antarctic by Leihy et al. (2023) to derive a list of introduced alien species for our eight study islands. Of the introduced vascular plant species that were recorded on our islands, we only kept those that: (1) are recorded at species level; (2) are listed as ‘certain’ to occur on the island; and (3) are terrestrial (see Figure 1 for the numbers per island and Data S2 the full list of species). We subsequently standardised species names according to the GBIF backbone checklist with the use of the R package ‘rgbif’ (Chamberlain et al. 2023). We downloaded all records of these species (*n* = 151) from the Global Biodiversity Information Facility (GBIF 2023). After which, we filtered out records that were recorded prior to 1981, as well as those with a coordinate uncertainty of more than 1 km. Fossil records and machine observations are not included in this study. Following this, we used the R package ‘CoordinateCleaner’ to omit records with equal longitude and latitude and those within a 0.5° radius of 0/0 coordinates, as well as records within 10 km radius of a country's capital and 1 km radius of a country's centroid. Lastly, we omitted observations within a 100 ‐m radius of biodiversity institutions and a 0.5° radius around the GBIF headquarters (Zizka et al. 2019). Notably, we did not differentiate between native and alien occurrences to ensure that the full realised niche of the species (excluding the islands) is captured. A total of 148 individual plant species remained after cleaning.

We aggregated species records located within the same 1 km grid cell of the climate layers and centred their coordinates to the centre of each cell. We subsequently thinned the records to 10 km with the use of the ‘spThin’ package (Aiello‐Lammens et al. 2015). Species with more than 65,000 records (*n* = 52) were thinned outside of the ‘spThin’ R package due to computational issues.

### 2.4 Species Distribution Modelling

#### 2.4.1 Preparation of Presence and Pseudo‐Absence Data

We performed the species distribution modelling (SDM) within the framework of the ‘biomod2’ R package (Thuiller et al. 2023). We randomly sampled as many pseudo‐absences as there were thinned occurrences, but only in continents where the species had more than 10 records after thinning. We weighted pseudo‐absence draws by the GBIF bias index of Meyer et al. (2015), in order to control for over‐representation of specific regions in global species occurrence data. We assigned the mean bias value of Australia to New Zealand, as the data for this country was absent in the data of Meyer et al. (2015). For species with less than 100 occurrences, the number of pseudo‐absences per draw was set to 100. Pseudo‐absence draws were repeated five times.

#### 2.4.2 Preparation of Predictor Sets

A total of seven different sets of four bioclimatic variables (CHELSA; Karger et al. 2017) were used to model species distributions. For simplicity and comparability, we thereby focused on differences in descriptors of temperature but kept those describing precipitation constant. Hence, all seven sets contain the annual precipitation (BIO12), and the precipitation seasonality (BIO 15) to reflect the quantity and variability of precipitation in our study region. The other two bioclimatic variables in each set represent different combinations of descriptors of the temperature regime (see Data S1 for full names): BIO 1, 3, 12 and 15; BIO 1, 7, 12 and 15; BIO 2, 5, 12 and 15; BIO 2, 6, 12 and 15; BIO 3, 5, 12 and 15; BIO 5, 7, 12 and 15; and BIO 6, 7, 12 and 15. Strongly correlated variables (−0.7 < *r* < 0.7) were not combined into one set except in one case (BIO6 and BIO7, *r* = −0.74; Data S3).

### 2.5 Statistical Algorithms and Model Performance

We used four statistical algorithms for distribution modelling to relate species' presences to pseudo‐absences, namely, generalised linear models (GLMs); generalised additive models (GAMs); random forests (RF); and boosted regression trees (BRTs), keeping the standard model parameters of biomod2 for each of the algorithms. The models were calibrated by partitioning the presence data into a set of 70% of points as training data and 30% of points as evaluation data. For each of the modelling techniques, three replications with the 70%–30% split‐data criterion were done.

The predictive power of each of the models was evaluated by the true skill statistic (TSS) (Allouche, Tsoar, and Kadmon 2006). The two regression methods (GLM and GAM) and two machine learning methods (RF and BRT) were used in ensemble modelling as implemented in biomod2 (Araújo and New 2007; Thuiller et al. 2023). Consensus models were made by combining all individual models with a TSS over 0.5. Ensemble predictions of species' occurrence probability were finally translated into binary presence‐absence predictions by using the threshold that maximised the TSS score. We are aware that for pseudo‐absence‐based models, statistics like AUC and TSS give a biased estimate of model performance (e.g. Lobo, Jiménez‐Valverde, and Real 2008). However, for comparing different models for the same species in the same study area, they are still a valid tool (Soley‐Guardia, Alvarado‐Serrano, and Anderson 2024).

### 2.6 Predictions

The binary output of the ensembles was used to predict occurrences of included species under current climatic conditions on the sub‐Antarctic islands. Island land surfaces were isolated from the GSHHG land mass shapefile at the highest resolution (Wessel and Smith 1996). For each 1 × 1 km^2^ grid cell that covers the islands' land surface, we determined if a species is projected to find suitable conditions there according to the model. We subsequently calculated the average percentage of cells per island (group) in which the species is predicted to occur.

To determine what proportion of the variance among SDM predictions can be explained by using models with different variable sets, we ran a factorial analysis of variance (ANOVA) with four groups and 3 two‐way interaction terms. The dependent variable was the amount of cells projected to be suitable for a species by the SDM. Included sources of variation were the total cell count of an island as a standardisation, the island's identity, the species' identity and the model (= predictor set) used. The 3 two‐way interaction terms were specified between the latter three variables. The contribution of each predictor (*η* ^2^) was calculated as a percentage by dividing the sum of squares (SSQ) of the respective predictor by the total SSQ across all predictors.

Finally, SDM predictions were evaluated by calculating the sensitivity and specificity for each combination of island and model by comparing the recorded species occurrences of Leihy et al. (2023) with the species predicted by our models. An island was considered suitable for a given species when the model predicted it in at least one of the island cells. The sensitivity is derived by dividing the ‘true positives’ (i.e. correctly predicted occurrences) by the sum of true positives and ‘false negatives’ (i.e. wrongly predicted absences). Specificity is calculated by dividing the number of ‘true negatives’ (i.e. correctly predicted absences) by the sum of true negatives and ‘false positives’ (i.e. wrongly predicted presences). An island‐ and model‐specific TSS value is subsequently derived by adding these values, minus one.

### 2.7 Niche Overlap Analysis

The climatic niche of each plant species realised outside of the sub‐Antarctic islands was determined by extracting the raster cell values containing thinned species occurrences from the bioclimatic grid layers provided by CHELSA (Karger et al. 2017). Similarly, we derived the climatic boundaries (i.e. minimum and maximum value across the island) of the individual sub‐Antarctic islands from the full set of grid cells that covers their land surface area. We treated all islands within an archipelago as one group, except the Crozet archipelago and Heard and McDonald islands, for which only records of the main Possession and Heard Island were available. All cell value extracting and spatial processing was done with the R package ‘terra’ (Hijmans 2023).

For all bioclimatic variables separately, we compared the climatic niche boundaries of each alien plant species outside of the sub‐Antarctic islands to the climatic range of each of the sub‐Antarctic islands on which they are present. We thereby calculated the percentage of occurrence records of a species (outside the sub‐Antarctic islands) that have bioclimatic conditions falling within the full climatic range of each respective island.

Finally, we tested whether predictor sets with higher weighted overlap between the conditions on an island and those in the species' range outside of the island will systematically project a higher suitability of the island for the respective species. First, we calculated for each species on each island an overlap metric by multiplying each bioclimatic variable's overlap value with the variable importance in the respective SDM and subsequently summing these weighted single‐predictor overlaps. Following this, we determined for which of the seven models the average overlap across all species and islands was the highest. Using that model as a baseline, we calculated a log‐response ratio (LRR) for each of the other six models as follows:

$$\ln\left(1+\frac{\left(X2-X1\right)}{X1}\right)$$

*X*1 is the proportion of island cells predicted to be suitable for a species by the baseline model. *X*2 is the proportion of cells on the same island predicted to be suitable for the same species by the model to be compared with the baseline. We used LRRs because they are a common measure of proportional change with desirable statistical properties (Hedges, Gurevitch, and Curtis 1999). We added a small constant (0.00001) to each proportion (*X*1 and *X*2) to avoid division by zero. Similarly, we calculated a second LRR to compare the same baseline model with the six other models in terms of the weighted cumulative overlap of the variables used in each predictor set. As a result, we got 142 (species) × 8 (islands) × 6 (comparisons to baseline model) LRRs for both the overlap in climate conditions between island and species range and the proportion of cells projected to be suitable for the focal species on the focal islands. To these two sets of LRRs, we finally fitted a linear mixed effects regression model with the LRR of cells projected suitable as the response, the LRR of climatic overlap as an explanatory variable and island and species as unnested random variables. All analyses were performed using R Statistical Software (v4.2.3; R Core Team 2021).

## 3 Results

### 3.1 Species Distribution Models

Of the 148 species included in our study, ensemble modelling was successful for 142 of them. Ensemble modelling of six species failed as no individual model had a TSS over 0.5 for at least one of the combinations of predictor variables.

**FIGURE 2:**
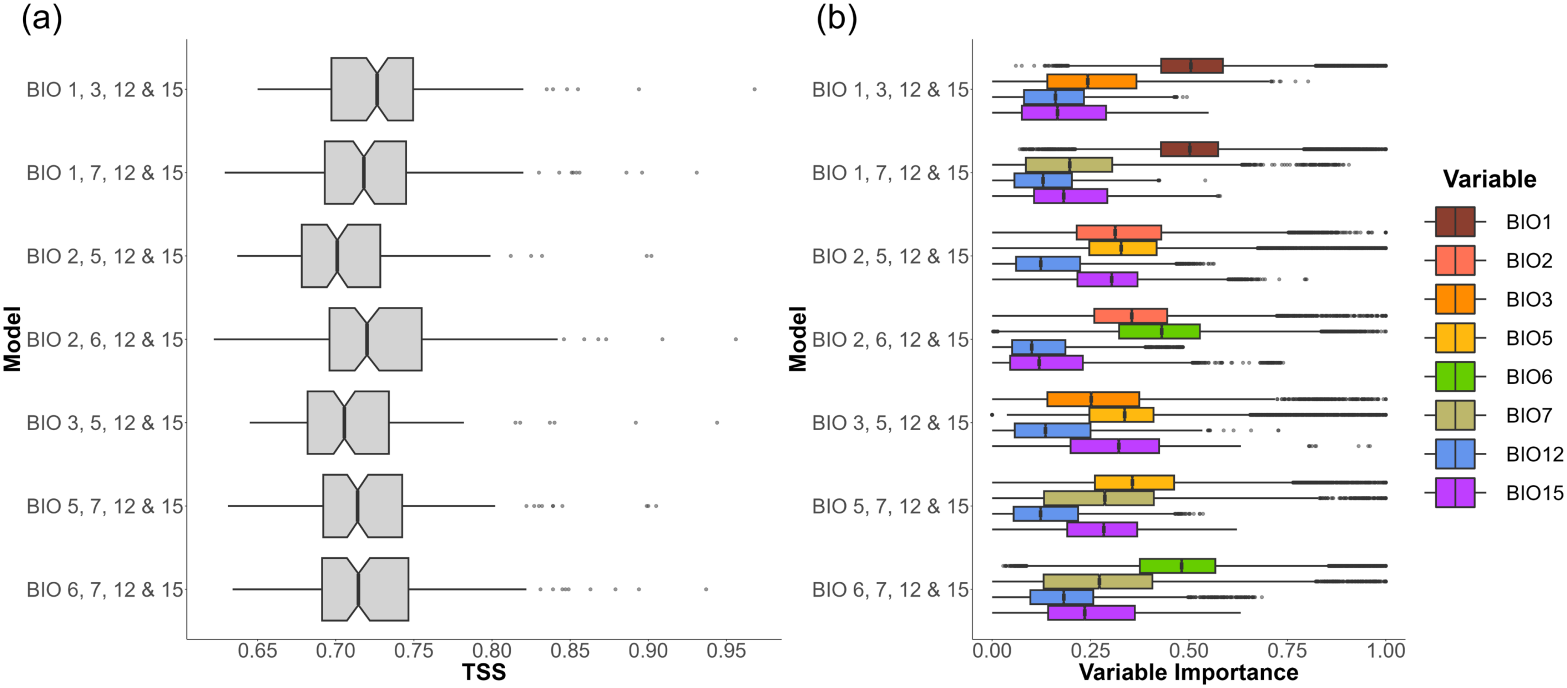
(a) TSS‐values of individual species' ensemble models per bioclimatic predictor set (TSS = sensitivity + specificity—1). (b) Mean variable importance across all species' SDMs for each of the predictor sets.

Overall, our models had good performances, with an average TSS across species of individual models used in the ensemble model of around 0.72 (Figure 2a). Models with mean annual temperature (BIO1) performed slightly better than average, while models using the mean daily maximum temperature of the warmest month (BIO5) did slightly worse. Nevertheless, the differences in model evaluation scores among the seven predictor sets were small. Mean TSS values of each individual model and species are provided in Data S4.

Bioclimatic variables related to temperature were generally more influential on the SDMs as compared to those relating to precipitation (Figure 2b; Data S5). In the two models that included mean annual temperature (BIO1), it was clearly the most important variable for the majority of species. Similarly, the mean maximum temperature of the warmest month (BIO5) and the minimum temperature of the coldest month (BIO6) were on average the most important variables in models wherein they were included. However, in models where BIO5 was included, the precipitation seasonality (BIO15) was also an influential variable.

### 3.2 Variability of Projections Onto the Islands Among Predictor Sets

**FIGURE 3:**
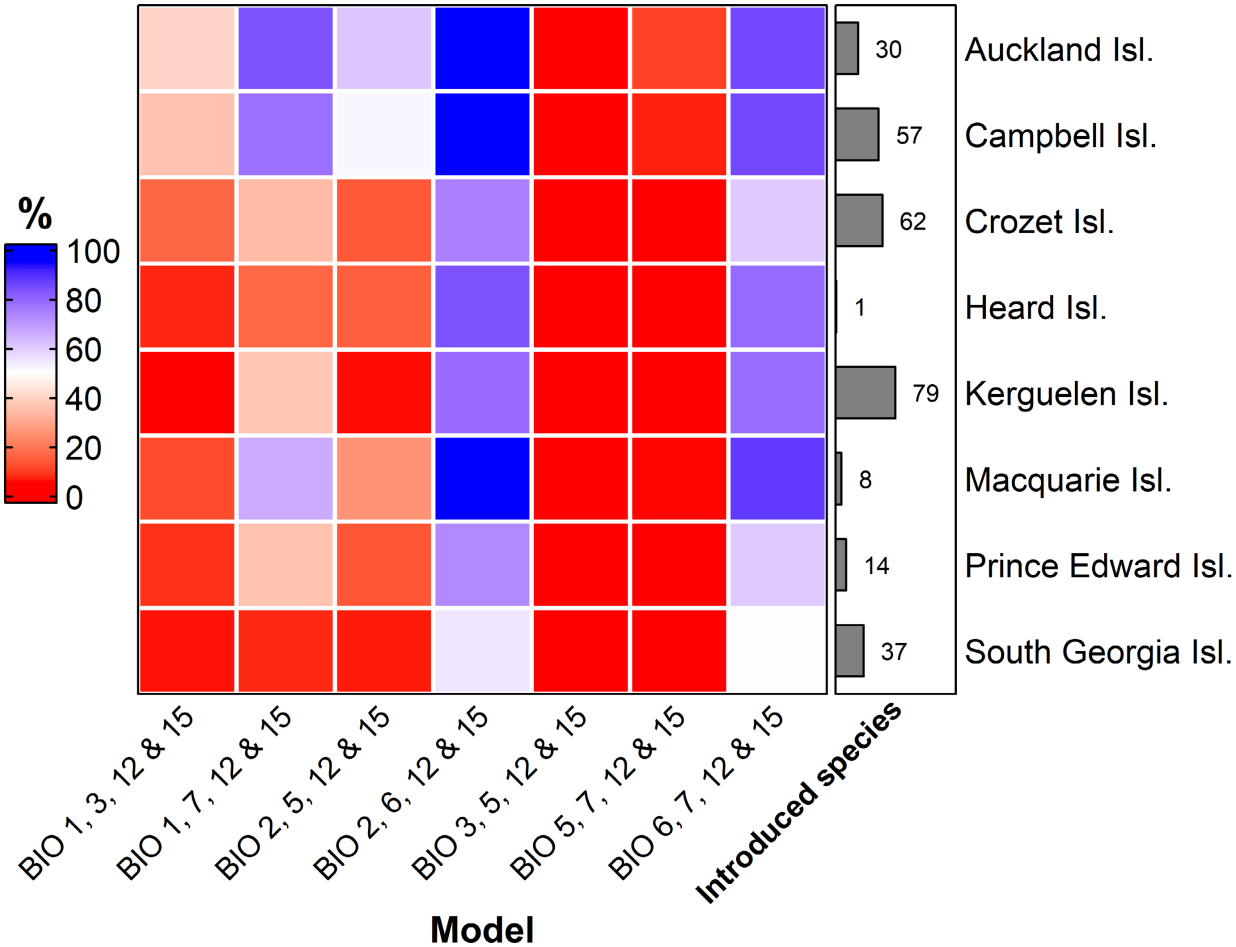
Heatmap showing the average percentage of island cells a species is predicted to occur in, averaged across all species per island, for each of the SDM models (= predictor sets). The amount of introduced species on each of the island (groups) included in the study (*n* = 142) is shown on the right side of the heatmap (Leihy et al. 2023; Data S2). Figure made with the ‘ComplexHeatmap’ R package (Gu, Eils, and Schlesner 2016).

**FIGURE 4:**
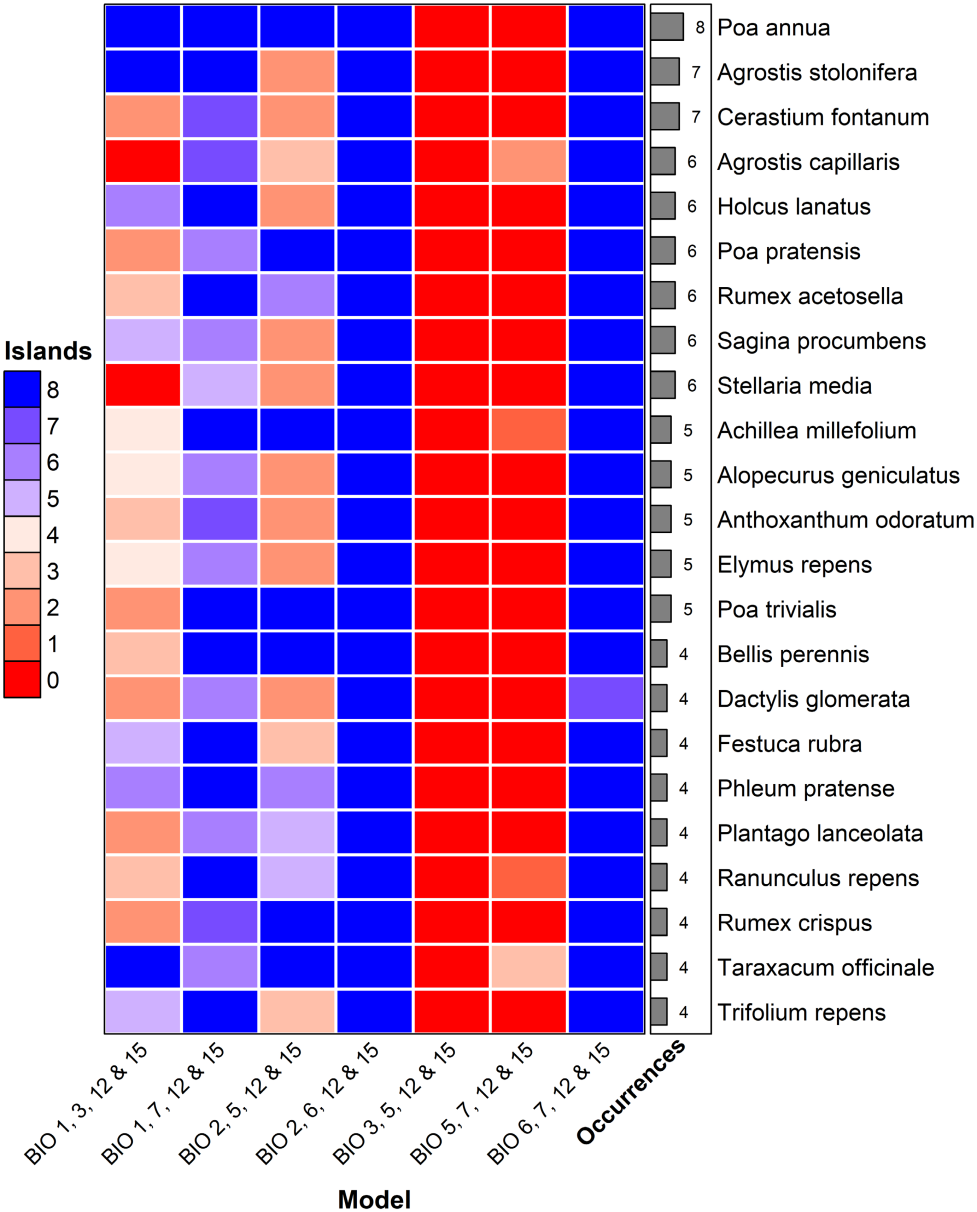
Heatmap showing the number of islands each species is predicted to occur on for each of the SDM models with different predictor variables. If a species is predicted on a single or more of the island cells, the species is considered to be predicted on the island. Included are all species that are currently occurring on four or more islands in the sub‐Antarctic, according to Leihy et al. (2023) (Data S2). The amount of island the species occurs on is shown on the right‐hand side of the heatmap. Figure made with the ‘ComplexHeatmap’ R package (Gu, Eils, and Schlesner 2016).

Projections of the seven different models onto the sub‐Antarctic islands differed vastly (Figures 3 and 4; see Data S6 for predicted percentage of island cells per species and model). While some of the models predicted almost no species to occur on any island, others projected almost all species onto each island or onto almost each individual cell on each island. As a corollary, variation in model projections was not only strong on average, but strong and consistent across almost all species (Figures 3 and 4). In addition, variation was not random among predictor sets. Across all species and islands, the number of cells predicted to be suitable varied between 101,928 and 2,924,056, which is 2.59% and 74.22% of total cells for all species and islands combined, respectively. Per species, the number of cells predicted suitable varied by 21,650 on average (SD = 6669.4). The three models containing BIO5 predicted an average of only 2.8%, 5.2% and 23.8% of all the island cells to be suitable for the 142 species. By contrast, the two models containing BIO6 projected the species onto 73.4% and 81.5% of the island cells, on average. Those containing BIO1 (mean annual temperature) projected species onto an intermediate amount of island cells, with 16.1% and 44.7% being the average across all species.

Despite their high accuracy when evaluated against random splits of the parameterisation data, all models performed poorly in predicting the occurrence of alien species on the sub‐Antarctic islands (Table 1; Data S7). Sensitivity was very high across all islands for the model with BIO2, 6, 12 and 15 (0.977), and the model with BIO6, 7, 12 and 15 (0.956), whereas their specificity was very low (0.067 and 0.121, respectively). Conversely, while the sensitivity of the model with BIO3, 5, 12 and 15 (0.955) and BIO5, 7, 12 and 15 (0.911) is high, their specificity is very low (0.018 and 0.055, respectively). This contrasting sensitivity and specificity translate into a very similar mean TSS for the seven models, all between −0.034 and 0.078. Although none of the predictor sets was clearly superior to the others in terms of TSS, those containing BIO5 tended to perform slightly worse than those containing BIO6 and BIO1 as temperature descriptors. However, as mentioned above, there was a clear split between models that were too liberal in projections and thus reaching high sensitivity but low specificity—especially those containing BIO6—and others that were too restrictive and thus reached high specificity but low sensitivity, especially those containing BIO5 (Table 1; Figure 3).

**TABLE 1:** Sensitivity, specificity and TSS values for each model and island (group), averaged across all species. Sensitivity is calculated by dividing the number of ‘true positives’, that is alien species occurrences correctly predicted by model, by the sum of ‘true positives’ and ‘false negatives’, that is alien species occurrences falsely not predicted by model. Specificity is derived by dividing the number of ‘true negatives’, that is species correctly predicted as absent, by the sum of true negatives and false positives, that is absent species falsely predicted as occurring by the model. The true skills statistic (TSS) is: Sensitivity + specificity − 1. Alien species occurrences on the sub‐Antarctic islands are listed in Leihy et al. (2023) (Data S2). Values for all models and islands are listed in Data S8.

Model	Sensitivity	Specificity	TSS
BIO 1, 3, 12 & 15	0.374	0.668	0.042
BIO 1, 7, 12 & 15	0.688	0.385	0.073
BIO 2, 5, 12 & 15	0.328	0.661	−0.010
BIO 2, 6, 12 & 15	0.977	0.067	0.044
BIO 3, 5, 12 & 15	0.018	0.955	−0.027
BIO 5, 7, 12 & 15	0.055	0.911	−0.034
BIO 6, 7, 12 & 15	0.956	0.121	0.078

**FIGURE 5:**
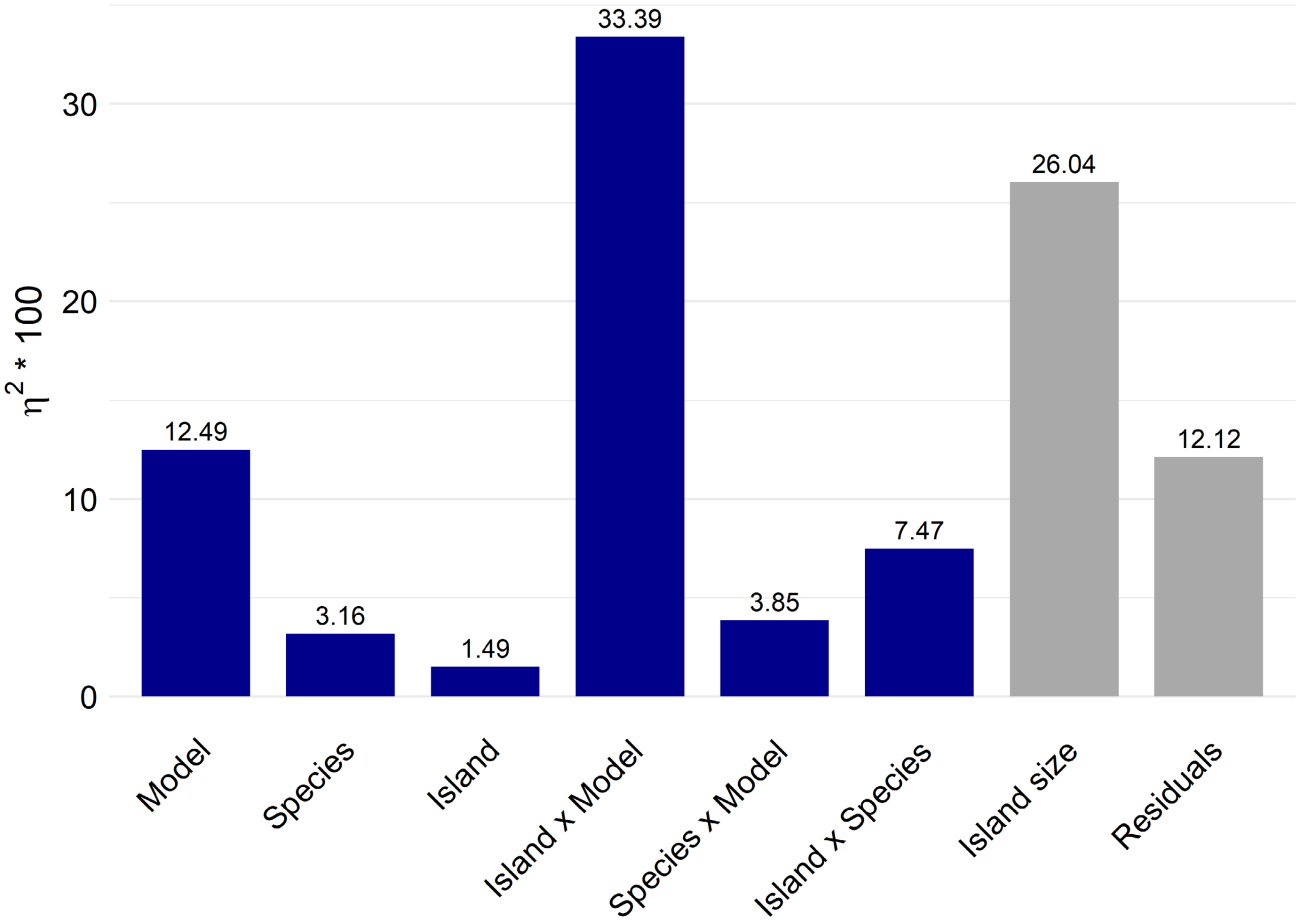
Variation in the amount of cells species is predicted by the SDMs that can be attributed to each factor, or two‐way interaction of factors, as well as the residual variance (*η* ^2^), determined from the ANOVA. Factors are the island's identity, species identity and the model (predictor set). Island size is included to standardise for the total amount of cells on each island. *η* ^2^ is calculated by dividing each factor's or interaction's sum of squares by the total sum of squares of all variables and their interactions combined.

When analysed in an ANOVA, of the variation in cells predicted to be suitable per island, 26% could be attributed to island size (i.e. total cell count), 1.5% to island identity, 3.2% to species identity, 12.5% to the model, that is the predictor set used, 7.5% to the interaction between island and species, 33.4% to the interaction between island and model, 3.9% to the interaction between species and model and 12.1% to further unknown sources of variation (Data S8; ANOVA table). Including the interaction terms, the selected model (= predictor set) hence was the most impactful source of variation in SDM projections (Figure 5).

### 3.3 Overlap of Species Distributions in the Sub‐Antarctic Islands With Climatic Variables

The proportion of overlap between the ranges of bioclimatic variables of invaded sub‐Antarctic islands with the climatic species' niches elsewhere varied substantially among individual islands (see Data S1 for climatic ranges of the islands and Data S9 for climatic ranges of species' realised niches). In general, the mean overlap between the islands' climatic conditions and the niches of its introduced species is largest for the Kerguelen Islands (44.2%, *n* = 79), Heard Island (30.5%, *n* = 1) and South Georgia (19.1%, *n* = 37) and very low on Campbell (8.4%, *n* = 57) and Crozet Island (7.4%, *n* = 62). The Auckland Islands (16.5%, *n* = 30), Macquarie Island (10.6%, *n* = 8) and Prince Edward Islands (10.5%, *n* = 14) have an intermediate percentage of overlap.

When comparing variables across islands, there is considerable overlap between species niches and the climate on all islands for some variables, but very low overlap with others (see Data S10 for mean overlaps and Data S11 for overlap per species and island). This variation is also represented among the variables used and/or varied in our predictor sets. Among the two precipitation variables contained in any model, overlap was relatively broad with respect to annual precipitation (BIO12, 25.9%), but low, on average, with respect to precipitation seasonality (BIO15, 3%). Among the varied temperature variables, it was high for isothermality (BIO3, 37%), and winter temperature (BIO6, 35.8%), intermediate for annual temperature (BIO1, 14.6%) and mean diurnal temperature range (BIO2, 5.7%), and very low for the temperature annual range (BIO7, 1.3%) and summer temperature (BIO5, 0.1%).

**FIGURE 6:**
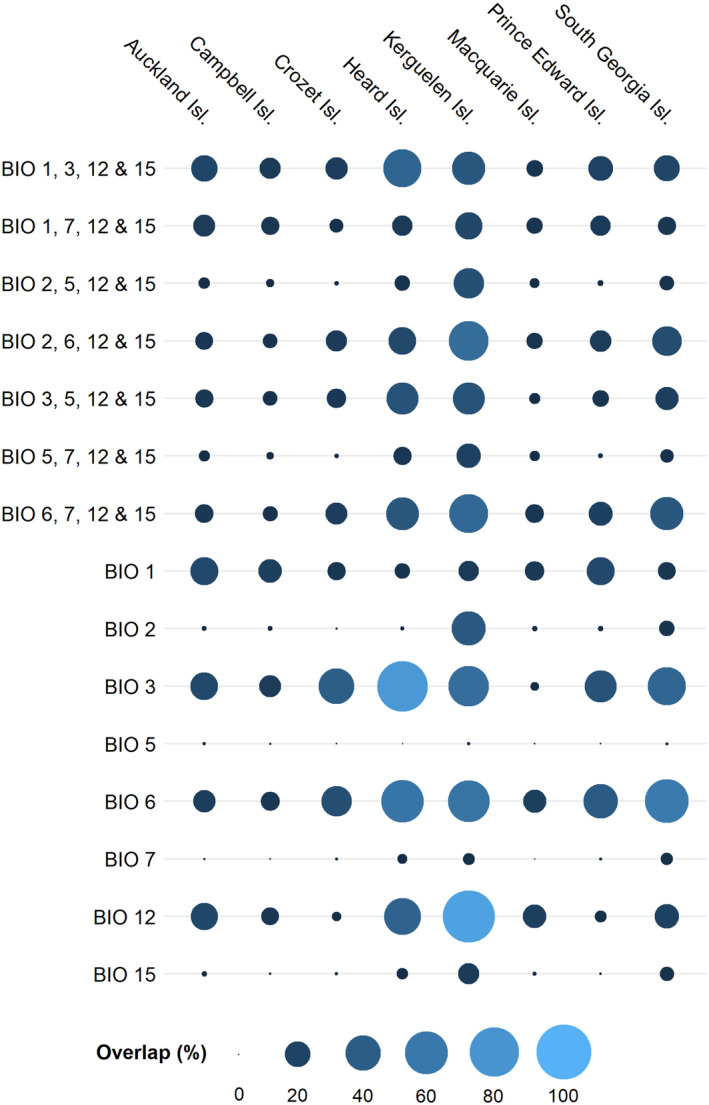
Cumulative percentage of overlap of four bioclimatic variables used in each predictor set, calculated by multiplying the variable importance of each variable with the percentage of overlap between climatic conditions (i.e. range of bioclimatic variables) of the sub‐Antarctic island group and the climatic niches of their alien flora realised outside the sub‐Antarctic islands. Additionally, the mean percentages of overlap are provided on a single‐variable basis (BIO1–BIO15). Note *n* = 1 for Heard Island. BIO1 = mean annual temperature, BIO2 = mean diurnal range, BIO3 = isothermality, BIO5 = maximal temperature of the warmest month, BIO6 = minimal temperature of the coldest month, BIO7 = temperature annual range, BIO12 = annual precipitation, BIO15 = precipitation seasonality.

The cumulative overlap of the four variables used in each predictor set, weighted by the variable importance of each predictor, largely reflects the patterns described above of single‐variable overlap (Figure 6; Data S12). Models with BIO5 included also show low overlap when looking at the variables combined in a predictor set, whereas models with BIO6 showed a higher cumulative overlap across all four variables. The exception is the model with BIO3, 5, 12 and 15 that still has a relatively high cumulative overlap, despite the inclusion of BIO5.

We found that the overlap between island and species range in terms of the climatic predictors used in the model has a positive effect on the area projected to be suitable for a species on an island (fixed‐effects estimate of LRR of cells projected suitable vs. LRR of climatic overlap = 1.458, SE = 0.07, *p* < 0.001) (see Data S13 for LRR values of each species and island combination and Data S14 for results of the LMM). Selecting predictors with a higher overlap hence tends to increase the part of the island projected to be suitable. There is, however, considerable variation in this relationship between species (random‐effect estimate of species = 1.72, SD = 1.31) and islands (random‐effect estimate of island = 0.81, SD = 0.9). This variability also resulted in the model with BIO3, 5, 12 and 15 projecting less suitable cells than that with BIO2, 5, 12 and 15, despite a higher overlap in predictor ranges.

## 4 Discussion

Our results corroborate that, as expected, the climatic conditions on the islands broadly overlap with those in the ranges the species occupy outside of the islands in some respects but not in others, with the islands' summer temperatures and temperature annual ranges particularly marginal to the alien species that currently occur on them. The climate of the islands is hence non‐analogue in the double sense that conditions spatially separated otherwise coincide, such as mild winters and cold summers, and that, mostly as a consequence, conditions are beyond those occupied by most species anywhere else with respect to one or the other of these climatic features. As expected, these non‐analogue conditions result in huge variation of alien plant species projections across the sub‐Antarctic islands in dependence of predictor variable choice. The effect of predictor variable choice was even stronger than that of island and species identity, even if the islands vary in their climates (Figure 1) and the species, obviously, in their climatic niches: the set of 142 species modelled include both rather warmth‐demanding ones native to Mediterranean regions (e.g. *Aira praecox*, *Avena fatua*) and those tolerant of cool conditions, for example at higher elevations of temperate mountains (e.g. *Avenella flexuosa*, *Deschampsia cespitosa*). Most impactful was the interaction between model and island, probably because predictor sets vary widely in whether they assign low or high suitability to all or the vast majority of species on particular islands. In terms of risk assessment, this implies that models do not only differ in predicting which species would find suitable habitat on the islands, but that they also deviate widely in terms of how conservative they are on a specific island. As expected, the degree of ‘conservativeness’, that is the area projected as suitable on each island, depended on the magnitude of overlap in variable values between the islands and the areas the species occupy outside of the islands. In particular, for many species and islands, the use of summer temperature, and to a lesser degree, annual mean temperature, had the potential to invert projections compared to the use of winter temperature, that is from the islands being almost completely unsuitable to highly suitable across most of their area.

Associated with the strong variation in predictions among species was a poor performance of all models to predict the actual establishment of alien plants on the island, despite high evaluation scores on random splits of the (global) parameterisation data. Of course, SDMs are tools to predict suitability not occurrence. In the case of alien species, the latter depends on other important factors, especially propagule pressure (Lockwood, Cassey, and Blackburn 2005). Whichever low such pressure, which is likely the case for these extremely remote islands, successful establishment will also be strongly co‐determined by ‘random’ factors, in particular for stowaways that represent the vast majority of established alien species in sub‐Antarctica. Such ‘random factors’ could, for example be place and time of deposition of imported seeds, whether the particular weather conditions in the year of deposition were favourable to germination and early survival, whether the seeds had been ‘collected’ from populations better or less pre‐adapted to the conditions on the islands, the conditions the seeds were exposed to during the trip on the ship, etc. While these factors may explain a large fraction of poor model performance, the non‐analogue conditions probably contribute their part, too. In particular, the unusual combination of highly permissive warm winters and strongly selective cold summer temperatures on the island implies vast overpredictions if summer conditions are disregarded and strong underpredictions if they are accounted for. A possible way forward is to build ensemble predictions of different predictor sets. This might at least allow identifying the species most likely to find suitable conditions as those that are projected to be suitable by all or at least the majority of the models (Araújo and New 2007).

An obvious consequence of these findings is that the choice of climate predictor variables deserves more attention than it receives in many SDM applications. In the vast majority of cases, this choice cannot be based on mechanistic knowledge of cause‐effect relationships (e.g. due to lack of information) but remains a rather subjective selection from a set of standard bioclimatic descriptors. Some of these descriptors are highly correlated; hence, model evaluation statistics do not provide any means of distinguishing between them, making the specific choice apparently unimportant. However, as our results suggest in line with earlier case studies (Synes and Osborne 2011; Braunisch et al. 2013), variation in subsequent projections can nevertheless be overwhelming. It is therefore crucial that modellers include such choices into their sensitivity analyses more routinely and report them in their publications (Zurell et al. 2020), especially if they use models to project to non‐analogue climates. Modellers should also pay more attention to the fact that non‐analogue does not only mean extrapolation beyond the currently realised value range but also involves changes to correlation structures. Such altered correlations are likely under changing climates (Braunisch et al. 2013), but, as our results emphasise, they might also be relevant when models are applied to predict suitability in other regions. The latter is standard in applications assessing the risk of alien species establishment or invasion (Thuiller et al. 2005; Dullinger et al. 2017; Haeuser et al. 2018) or in designing conservation management strategies (Guisan et al. 2013). Especially cross‐continental invasion risk assessments often involve projections into non‐analogue climates, including those where some of the variables show little overlap between parameterisation and projection range (Early and Sax 2014).

The emphasis on predictor choice shall not downplay the effects that other methodological decisions may have on model projections. The selection of modelling algorithms is probably the one decision with the largest such effect (e.g. Guisan et al. 2007; Thuiller et al. 2019). When it comes to projections onto non‐analogue conditions, the decision for one or the other algorithm might even become more critical as these algorithms are known to differ in how they extrapolate beyond the value range captured by parameterisation data (Elith, Kearney, and Phillips 2010). In our results, the higher suitability projected by the variable set with BIO2, 5, 12 and 15 as compared to BIO3, 5, 12 and 15, despite lower predictor overlap of the former, could result from such differences. Plant species distributions have recently been shown to be more sensitive to strong diurnal (BIO2) than annual (BIO3) temperature variability (Gallou et al. 2023). Therefore, many species will probably perform well in areas with small diurnal temperature ranges, resulting in tree‐based algorithms such as RF and GBM to extrapolate high suitability to even smaller such ranges as realised on the sub‐Antarctic islands. In addition, what has been explored less well is that these models also differ in how they handle predictor interactions, which are implicitly accounted for in regression tree‐based methods but commonly neglected with algorithms such as GLMs or GAMs. Consequently, they might also be differentially sensitive to non‐analogue climates in the sense of changed correlation structures. Accordingly, predictor and algorithm choice in combination have considerable potential to induce even stronger variation, and hence uncertainty, in model projections to all kinds of non‐analogue conditions.

Pronounced variation among model projections in dependence on predictor sets also has implications for studying possible niche shifts, for example between native and introduced ranges or between parts of the native range (e.g. Petitpierre et al. 2012; Wasof et al. 2015). Indeed, whether alien species typically maintain their climatic niches while dispersing into new habitats or not is still contentious (Gallagher et al. 2010; Petitpierre et al. 2012; Early and Sax 2014; Tingley et al. 2014; Capinha et al. 2018; Liu et al. 2020a, 2020b). In our case study, many alien species have been found on many islands, which some of the models have classified as unsuitable and some as suitable to them. As a consequence of this fact, the conclusion on whether the realised climatic niches of the species have changed or not would depend on the predictor set used for modelling. The common use of latent variables, such as PCA axes, in range shift studies (Broennimann et al. 2012) reduces the problem because collinear variables can be included and subjective predictor choice can be avoided. However, the result is a ‘compromise’ that depends on how the predictors score on the latent variables. Clarity can be provided by a full screening of niche overlap along various dimensions of the climatic niche as we have performed here. The results demonstrate that the realised niche of many aliens has indeed expanded, or shifted, with respect to several climatic variables, especially summer warmth. However, the mechanistic interpretation of these shifts remains unclear: we cannot distinguish whether the species had to adapt to low summer temperatures for successful colonisation or whether they would always have been able to tolerate cold summers given that they would have been combined with other climatic aspects in the way they are on the islands elsewhere across their range. In fact, the geographical isolation of the sub‐Antarctic islands suggests that dispersal limitation has been an important constraint on their colonisation; and that species successful today may not have been restricted by climatic conditions before humans introduced the species. In physiological terms, the mild winter temperatures may reduce the need for investments into frost adaptation and thus decrease the need for thermal energy in summer. Moreover, the widespread distribution of many plants that invaded the sub‐Antarctic islands suggests that these species are generally cosmopolitan and able to grow under a broad array of environmental conditions and have high phenotypic plasticity, a factor known to facilitate successful establishment under novel environmental conditions (Sexton et al. 2009; Davidson, Jennions, and Nicotra 2011; Fristoe et al. 2021). Further, the long‐lasting isolation and small native flora of the sub‐Antarctic islands may have facilitated invasions through unused niche opportunities (Moser et al. 2018). Finally, the invasion of entirely novel plant species from the opposite hemisphere happened alongside the introduction of novel faunal aliens (e.g. mice, rats and rabbits), with which these species share an eco‐evolutionary history. As a result, the alien flora had the opportunity to evolve adaptations mitigating the impacts of this fauna, whereas the native sub‐Antarctic flora did not. These latter aspects may have fostered successful alien establishment even if climatic conditions were only marginally suitable to the newcomers.

## 5 Conclusions

The need to better protect biodiversity is increasingly recognised, and ambitious international agreements on expanding and restoring intact ecosystems and halting biological invasions have recently been reached (CBD 2022). Therefore, the demand for biodiversity scenarios and their impact on management decisions will likely continue to increase in the future. Understanding and reporting the uncertainties associated with these scenarios is therefore crucial. While some of these uncertainties, especially those linked to modelling algorithms, dispersal assumptions, climate scenarios and GCMs, are now routinely included in SDM‐based scenarios, those linked to predictor choice are not. The results presented here clearly underline that this is vital.

Our findings also provide an example of how the uncertainty of predictions compounds when an SDM is used to project into novel environmental space. The sub‐Antarctic islands present a rare example of a climate with unique characteristics compared to the rest of the world. Our results highlight the challenges of modelling species distributions under such circumstances. The transferability of SDMs that rely on extensive extrapolation through time or space has been argued (Peterson 2011; Maguire et al. 2016). For example, SDMs have been found to poorly predict present day distribution of plant species when calibrated on distributions on late glacial fossil‐pollen data, even though they predict distribution of those species in the late glacial period well (Veloz et al. 2012). Novel conditions are increasingly likely to emerge under climate change (Fitzpatrick and Hargrove 2009). This would introduce rising uncertainty in model predictions as extrapolation increases, especially in areas that currently occupy the warm margins of global climate. Therefore, this source of uncertainty should receive special attention in reports of SDMs that rely on extrapolation of the climatic niche.

With respect to the special case of biological invasions on the sub‐Antarctic islands, our findings demonstrate that these species frequently grow there under climatic conditions that are in several aspects different from the conditions they experience anywhere else in the world. Unique climates hence do not buffer regions against alien species, and conversely, at least with respect to some dimensions of these niches, shifts of realised climatic niches can occur frequently.

## Author Contributions

**Tom Vorstenbosch:** conceptualization (equal), formal analysis (lead), writing – original draft (lead), writing – review and editing (lead). **Franz Essl:** writing – original draft (supporting). **Bernd Lenzner:** formal analysis (supporting), writing – original draft (supporting). **Johannes Wessely:** formal analysis (supporting), writing – original draft (supporting). **Stefan Dullinger:** conceptualization (equal), formal analysis (supporting), funding acquisition (lead), writing – original draft (supporting), writing – review and editing (supporting).

## Conflicts of Interest

The authors declare no conflicts of interest.

## Supporting information


Data S1.

## Data Availability

The data that support the findings of this study are openly available in Dryad at 10.5061/dryad.9ghx3ffqd.
