# Supplementary material for: Venturing Into the Unknown: The Importance of Variable Selection When Modelling Alien Species Under Non‐Analogue Climatic Conditions
Source: Ecol Evol. 2024 Oct 28;14(10):e70490. doi: 10.1002/ece3.70490 (PMC11518623; doi:10.1002/ece3.70490)
Supplement: Supplementary file 1 — Data S1. [file ECE3-14-e70490-s001.zip › S1.docx]

**S1.** The value ranges (Minimum – Maximum) of 19 bioclimatic variables for the sub-Antarctic islands, during the period 1981 to 2010 (CHELSA; Karger et al., 2017).

|  | Auckland Islands | Campbell Islands | Crozet Islands (Possession Isl.) | Heard Island | Kerguelen Islands | Macquarie Island | Prince Edward Islands | South Georgia Islands |
| --- | --- | --- | --- | --- | --- | --- | --- | --- |
| BIO1 **- Mean annual temperature (°C)** | 5.25 – 8.45 | 5.05 – 7.45 | 2.85 – 5.35 | -11.45 – 1.75 | -2.45 – 4.25 | 3.15 – 5.15 | 1.35 – 5.95 | -12.35 – 1.45 |
| BIO2 **- Mean diurnal range (°C)** | 1.9 – 2 | 1.9 – 2 | 2.3 – 2.3 | 1.8 – 1.9 | 2.1 – 6.3 | 2.1 – 2.2 | 2.4 – 2.5 | 1.8 – 3.5 |
| BIO3 **– Isothermality** | 33.1 – 41 | 34.5 – 39.5 | 29.8 – 39.9 | 16.2 – 36.5 | 27.5 – 50.1 | 37.4 – 39.8 | 30.9 – 44.6 | 23.9 – 35.2 |
| BIO4 **- Temperature seasonality (St. dev.)** | 109.5 – 149.1 | 113.7 – 141.8 | 121.7 – 184.4 | 111 – 317.1 | 115.7 – 285.2 | 122 – 135 | 106 – 181.6 | 150.5 – 344 |
| BIO5 **– Maximum temperature of warmest month (°C)** | 8.55 – 11.15 | 8.15 – 10.15 | 6.95 – 8.35 | -5.15 – 4.25 | 2.95 – 10.25 | 6.25 – 8.15 | 5.55 – 8.95 | -6.35 – 5.45 |
| BIO6 **– Minimum temperature of coldest month (°C)** | 2.55 – 6.25 | 2.55 – 5.15 | -0.75 – 2.55 | -16.65 – -0.85 | -8.55 – 1.35 | 0.55 – 2.65 | -2.55 – 3.15 | -19.45 – -1.55 |
| BIO7 – **Temperature annual range (°C)** | 4.9 – 6 | 4.9 – 5.6 | 5.7 – 7.7 | 5 – 11.5 | 5.4 – 13.2 | 5.5 – 5.8 | 5.7 – 8.1 | 6.1 – 13.1 |
| BIO8 **– Mean temperature of wettest quarter (°C)** | 6.95 – 9.85 | 5.15 – 7.45 | 0.85 – 4.05 | -14.55 – 2.25 | -5.45 – 5.05 | 4.35 – 6.15 | 2.05 – 6.45 | -10.65 – 3.85 |
| BIO9 **– Mean temperature of driest quarter (°C)** | 4.15 – 7.85 | 3.55 – 6.15 | 5.45 – 7.05 | -12.45 – 1.05 | -2.75 – 7.15 | 2.25 – 5.05 | 0.85 – 5.25 | -10.05 – 1.55 |
| BIO10 – **Mean temperature of warmest Quarter (°C)** | 7.55 – 10.25 | 7.25 – 9.15 | 5.65 – 7.15 | -6.75 – 3.35 | 1.45 – 7.15 | 5.25 – 7.15 | 3.95 – 7.55 | -7.75 – 4.15 |
| BIO11 – **Mean temperature of coldest Quarter (°C)** | 3.65 – 7.25 | 3.55 – 6.15 | 0.55 – 3.85 | -15.45 – 0.35 | -5.75 – 2.55 | 1.75 – 3.95 | -1.15 – 4.55 | -17.45 – -0.45 |
| BIO12 – **Annual precipitation (mm/year)** | 964.8 – 1512.4 | 1260.9 – 1600.7 | 2165.2 – 4474.8 | 848.3 – 3287 | 599.3 – 5123.1 | 999.8 – 1394.1 | 2049.4 – 4957.2 | 1328.1 – 6553.5 |
| BIO13 – **Precipitation of wettest month (mm/month)** | 94.8 – 149.7 | 120.5 – 152.6 | 198.9 – 411 | 84.7 – 325.5 | 59.6 – 508 | 99.3 – 139.2 | 191.9 – 458.3 | 133 – 678.5 |
| BIO14 – **Precipitation of driest month (mm/month)** | 70.6 – 111.3 | 93 – 118.7 | 147.5 – 302.4 | 56.9 – 214.6 | 38.7 – 341.9 | 73.9 – 102.7 | 146.8 – 356.3 | 94 – 521.4 |
| BIO15 – **Precipitation seasonality (coefficient of variation)** | 8.8 – 9.9 | 7.8 – 8.1 | 7.4 – 8.4 | 14.4 – 16 | 12.8 – 17.1 | 8.7 – 9.4 | 7.3 – 8 | 8.6 – 12.4 |
| BIO16 – **Precipitation of wettest quarter (mm/quarter)** | 269.2 – 422.8 | 352.2 – 447.8 | 586.7 – 1216.5 | 246 – 959.3 | 173.8 – 1487 | 287.5 – 403 | 574.1 – 1368.4 | 389.2 – 2027 |
| BIO17 – **Precipitation of driest quarter (mm/quarter)** | 216.5 – 342 | 283.6 – 359.4 | 459.4 – 941.5 | 173.1 – 662 | 122.3 – 1038.1 | 226.9 – 316.6 | 451.4 – 1089.9 | 282.2 – 1571.1 |
| BIO18 – **Precipitation of warmest quarter (mm/quarter)** | 238.4 – 375.7 | 297.8 – 378 | 520.7 – 1065 | 230.4 – 879.4 | 128 – 1384.3 | 257.8 – 358.4 | 510.3 – 1208.3 | 372.3 – 1822.8 |
| BIO19 – **Precipitation of coldest quarter (mm/quarter)** | 228.6 – 364.6 | 283.6 – 365.6 | 586.7 – 1212.2 | 205.4 – 825.4 | 147.3 – 1215.9 | 243.6 – 333.8 | 509.4 – 1254 | 300.6 – 1736.2 |
