# Supplementary material for: Venturing Into the Unknown: The Importance of Variable Selection When Modelling Alien Species Under Non‐Analogue Climatic Conditions
Source: Ecol Evol. 2024 Oct 28;14(10):e70490. doi: 10.1002/ece3.70490 (PMC11518623; doi:10.1002/ece3.70490)
Supplement: Supplementary file 1 — Data S1. [file ECE3-14-e70490-s001.zip › S14.docx]

|  | *est.* | *SE* | *t* | *p* | Variance | *SD* | No. of obs. |
| --- | --- | --- | --- | --- | --- | --- | --- |
| **Fixed effects** | | | | | | |  |
| (Intercept) | -6.72301 | 0.35585 | -18.89 | < .001*** |  |  |  |
| Log response ratio (LRR) – overlap | 1.45761 | 0.07441 | 19.59 | < .001*** |  |  | 6816 |
| **Random effects** | | | | | | |  |
| Island |  |  |  |  | 1.724 | 1.3130 | 8 |
| Species |  |  |  |  | 0.809 | 0.8994 | 142 |
| Residual |  |  |  |  | 65.282 | 8.0798 |  |

**S14.** Results of the linear mixed model (LMM) to test the effect of the log-response ratio (LRR) of the overlap between species’ realized niche and islands’ climate on the LRR of prediction (i.e. the proportion of cells a species is predicted to occur in according to the SDMs). The model with the highest mean overlap (BIO6, 7, 12 & 15) across all species is used as a baseline for calculating LRRs. Island identity and species identity where used in the model as non-nested random factors.
