# Supplementary material for: Venturing Into the Unknown: The Importance of Variable Selection When Modelling Alien Species Under Non‐Analogue Climatic Conditions
Source: Ecol Evol. 2024 Oct 28;14(10):e70490. doi: 10.1002/ece3.70490 (PMC11518623; doi:10.1002/ece3.70490)
Supplement: Supplementary file 1 — Data S1. [file ECE3-14-e70490-s001.zip › S8.docx]

**S8.** ANOVA test table. The dependent variable in this analysis is the amount of predicted island cells by the SDMs. The four factors are island cell count (total cells on each island), island identity, species identity, and model (i.e. variable set used in the SDM). The degrees of freedom (Df), sum of squares (SS), mean sum of squares, *F*-statistic, *p*-values, and eta-squared (η^2^) are listed as well.

| **Variable** | **Df** | **SS** | **MSS** | **F** | **p** | **η^2^** |
| --- | --- | --- | --- | --- | --- | --- |
| Cell count | 1 | 1.689e+10 | 1.689e+10 | 12726.455 | < .001*** | 0.260 |
| Island | 6 | 9.643e+08 | 1.607e+08 | 121.102 | < .001*** | 0.015 |
| Species | 141 | 2.048e+09 | 1.452e+07 | 10.942 | < .001*** | 0.032 |
| Model | 6 | 8.099e+09 | 1.350e+09 | 1017.167 | < .001*** | 0.125 |
| Island:Species | 987 | 4.844e+09 | 4.908e+06 | 3.699 | < .001*** | 0.075 |
| Island:Model | 42 | 2.166e+10 | 5.157e+08 | 388.583 | < .001*** | 0.334 |
| Species:Model | 846 | 2.495e+09 | 2.949e+06 | 2.222 | < .001*** | 0.038 |
| Residuals | 5922 | 7.859e+09 | 1.327e+06 |  |  | 0.121 |
